# Case Report: Exploring delayed hyperprogressive disease: a case study of post-immunotherapy in lung cancer

**DOI:** 10.3389/fimmu.2025.1552547

**Published:** 2025-04-17

**Authors:** Jie Zhou, Kexin Cao, Jin-Xia Wei, Biao Wang, Meng-Jie Li, Jian Zhu, Guo-Ping Ai, Qiu-Lian Liu

**Affiliations:** ^1^ Department of Respiratory Oncology, Renmin Hospital of Qingxian, Cangzhou, China; ^2^ Department of Thoracic Surgery, The First Affiliated Hospital of Xinxiang Medical University, Xinxiang, China; ^3^ Department of General, General Hospital of Central Theater Command of the People’s Liberation Army, Wuhan, China; ^4^ Department of Thoracic Cardiovascular Surgery, General Hospital of Central Theater Command of the People’s Liberation Army, Wuhan, China; ^5^ Department of Radiology, General Hospital of Central Theater Command of the People’s Liberation Army, Wuhan, China; ^6^ Jiujiang City Key Laboratory of Cell Therapy, Department of Oncology, The First Hospital of Jiujiang City, Jiujiang, China

**Keywords:** lung cancer, immunotherapy, chemotherapy, radiotherapy, hyperprogression disease

## Abstract

**Background:**

Recent studies have shown that immunotherapy improves survival outcomes for patients with a late staged cancer. However, in a small number of cases do not benefit from this treatment and instead experience rapid tumor progression, known as hyperprogressive disease (HPD). Currently, HPD is provisionally defined as occurring within two months of receiving immunotherapy. Is HPD that occurs after two months associated with immunotherapy? The existing literature does not provide an answer.

**Case presentation:**

A 59-year-old woman was diagnosed with unresectable squamous cell carcinoma of the lung. She received four months (6 cycles) of chemotherapy with albumin-bound paclitaxel and cisplatin, along with immunotherapy using Camrelizumab. After treatment, the lesion in the patient’s lung were significantly reduced. However, because the tumor did not disappear and due to the limitations dose of the chemotherapy drugs using for body, the patient turned to receive stereotactic radiation therapy (2 Gy per fraction). After 10 fractions of radiotherapy, the lesion in the patient’s lung significantly increased. The enlarged lesion was pathologically analyzed through a percutaneous lung biopsy and was confirmed to be squamous cell carcinoma. Following the cessation of radiotherapy, four cycles of targeted segment arterial chemoembolization resulted in another significant reduction in the lung lesion.

**Conclusions:**

This report is the first to present HPD after 5 months of immunotherapy, marking the longest recorded occurrence of this phenomenon. This particular case of post-immunotherapy HPD achieved satisfactory results through targeted segment arterial chemoembolization, offering a potential approach for managing this side effect.

## Introduction

In recent years, immunotherapy has emerged as an important option for treating non-small-cell lung cancer (NSCLC) patients, with programmed death receptor-1/-ligand 1 (PD-1/PD-L1) immune checkpoint inhibitors (ICI) in combination with platinum-containing double-agent chemotherapy now established as the primary first-line therapeutic approach in clinical settings (1–3). While immunotherapy treatments are generally successful, immune-related adverse events (irAEs) do occur occasionally (4). Further, PD-1/PD-L1 blockage can result in an unsatisfactory response pattern characterized by tumor growth acceleration and a poor prognosis (5). And so, a phenomenon known as hyperprogressive disease (HPD) has been proposed, in which tumor growth becomes paradoxically accelerated after immunotherapy (6). The term “hyperprogression” was provisionally defined as an increase in tumor burden and twofold increase in tumor growth rate within 2 months following treatment failure (7). HPD’s definitional criteria and biological basis are not agreed upon, which necessitates multicenter studies and collaborations (5).

## Case presentation

**Figure 1:**
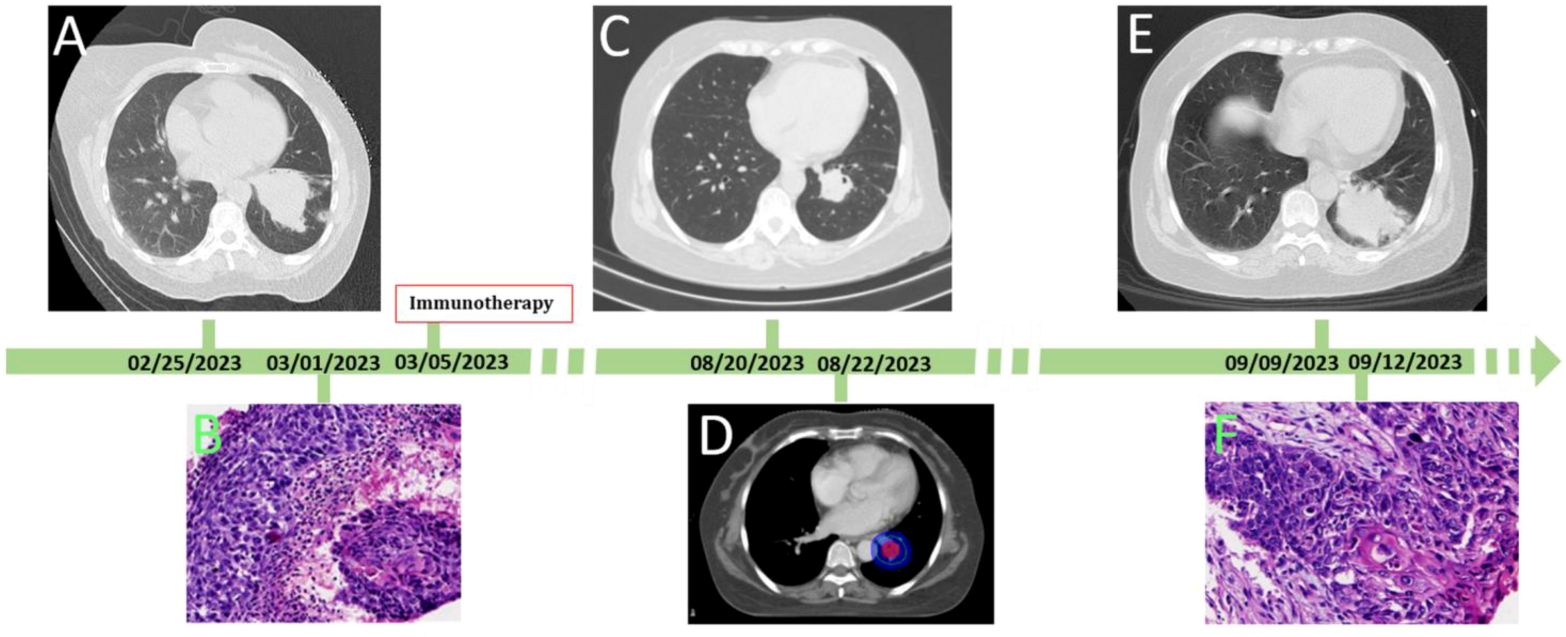
The process of the patient’ disease evolution and treatments. **(A)** at first diagnosis, chest CT showed the lesion size was 53 mm×52 mm; **(B)** histologic diagnosis the lesion was squamous cell carcinoma of lung; **(C)** after chemotherapy combined with immunotherapy, the lesion decreased at 23×25mm in size; **(D)** stereotactic conformal radiotherapy followed; **(E)** after 10 radiotherapy sessions, the patient developed dyspnea and fever, Reexamination of chest CT showed the lesion was significant enlargement at 50×51mm in size; **(F)** histologic diagnosis the enlargement lesion was squamous cell carcinoma of lung.

A 59-year-old woman presented with a cough and underwent a lung CT scan, which revealed a space-occupying lesion measuring 52×53 mm in the left lower lobe (**Figure 1A**). The lesion was accompanied by peripheral obstructive inflammation, atelectasis, and multiple enlarged lymph nodes in the mediastinum, located behind and below the left main bronchus, as well as adjacent to the left lower pulmonary vein. Lung cancer with hilar lymph node metastasis was diagnosed following consultation with a multidisciplinary team (MDT). The team concluded that R0 resection for this lesion was not possible. Following this, the patient underwent a lung puncture biopsy and was diagnosed with keratinized squamous cell carcinoma (**Figure 1B**). Immunohistochemical staining revealed positivity for CK5/6, P63, P40, and Ki-67 (with Ki-67 positivity at 10%), and negativity for Napsin A and CK7. Therefore, the patient was diagnosed with left lower lobe squamous cell carcinoma (T3N2bM0, IIIB, clinical stage). Subsequently, she received six cycles of albumin-bound paclitaxel (100 mg/m² on day 1, day 8, and day 15), cisplatin (75 mg/m² on day 1), and carrelizumab (200 mg on day 1) as determined by an MDT discussion. During this treatment, the patient experienced some common side effects, which were successfully alleviated with conventional methods. Throughout this process, the patient’s lung tumor gradually shrank. After completing six cycles of chemotherapy plus immunotherapy, a chest CT reexamination revealed that the left lower lung lesion had significantly reduced to approximately 23×25 mm (**Figure 1C**), while the lymph nodes remained visible.

After further consultation and discussion with the MDT, R0 resection was still not possible under the current conditions. However, because the tumor did not disappear and due to the limitations dose of the chemotherapy drugs using for body, the MDT’s second discussion concluded that the next appropriate treatment should be radiotherapy, with a dose of 2 Gy per fraction, administered over a total of 30 fractions. The patient then underwent target localization and planned radiotherapy (**Figure 1D**). However, after ten fractions of radiotherapy, the patient developed dyspnea and fever, with a maximum temperature of 38.5°C. Re-examination of the chest CT showed that the left lower lung lesion had significantly enlarged to a size of 50 × 51 mm (**Figure 1E**). After the third MDT discussion, the hyperprogressive disease of the lesion was considered first. Subsequently, a lung puncture biopsy of the enlarged part of the lesion was performed, and the pathological findings of the biopsy indicated squamous cell carcinoma (**Figure 1F**). Immunohistochemical staining was positive for CK5/6, P63, and P40, and negative for Napsin A, Syn, and TTF-1. At this stage, the delayed HPD after 5 months of immunotherapy was confirmed.

**Figure 2:**
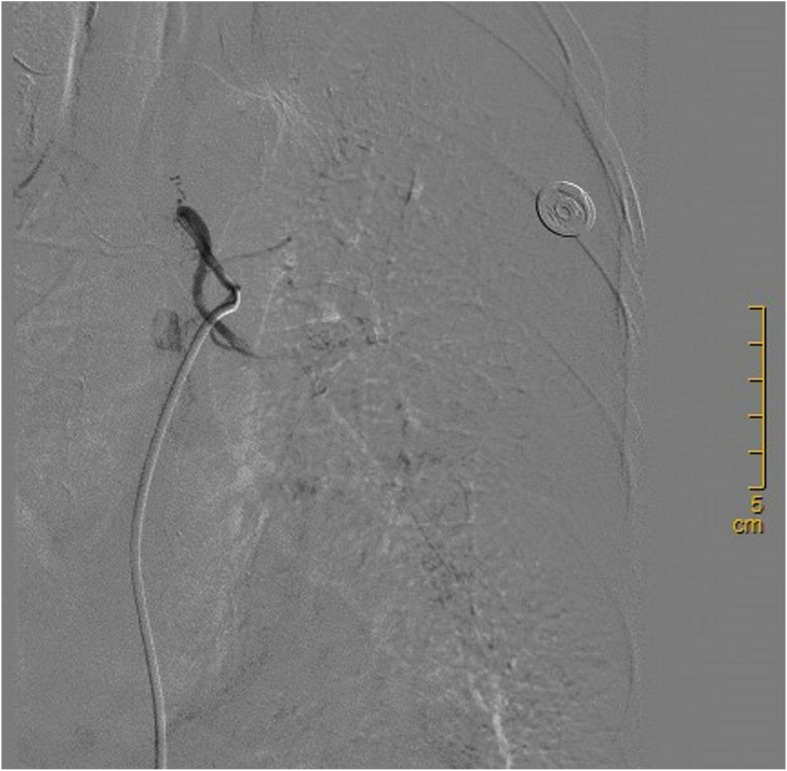
The lesion was received target arterial chemoembolization therapy.

**Figure 3:**
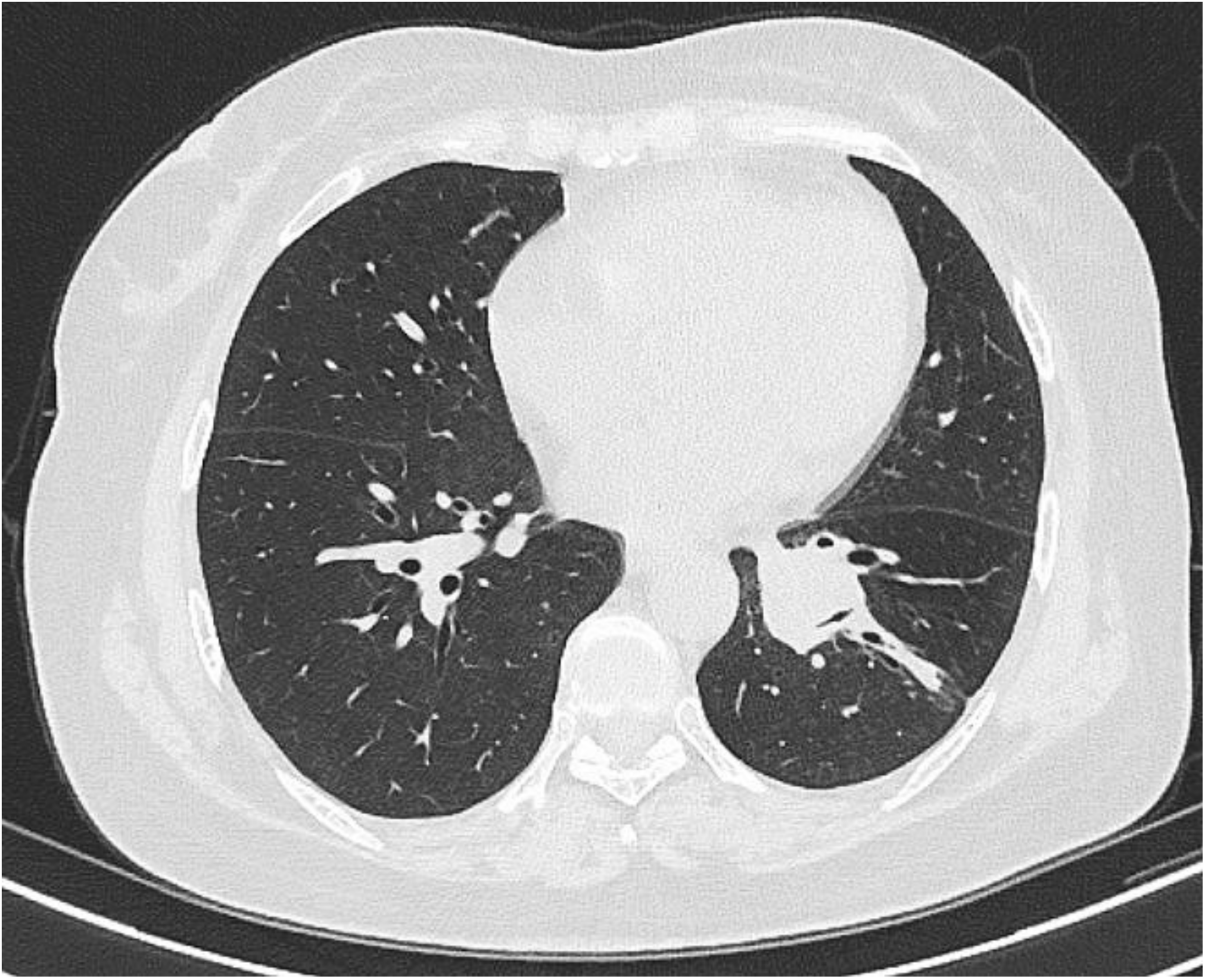
16-slice computerized tomography images of the reexamination of chest CT showed the lesion significant reduction.

Four cycles of target segment arterial chemoembolization were performed (100 mg of paclitaxel, 60 mg of cisplatin, and gelatin sponge embolization; see **Figure 2**). After completion of treatment, chest CT reexamination revealed that the lesion in the left lower lung had shrunk again, with a size of approximately 21×24 mm, and the enlarged lymph nodes in the mediastinum had basically disappeared (**Figure 3**). The patient expressed satisfaction with the doctor’s diagnosis and treatment. Then, she was received surgery at the outside hospital.

## Discussion

This case report describes a patient with unresectable non-small cell lung cancer who achieved a doubt reducing in the size of the lesion after treatment with immunotherapy using Camrelizumab. After five months of multiple treatment, the lesion in the patient’s lung significantly increased. The diagnosis of HPD was confirmed by pathology of the lung puncture biopsy for the increased part of the lesion. Following the cessation of radiotherapy, four cycles of targeted segment arterial chemoembolization resulted in another significant reduction in the lung lesion. This report is the first to present HPD after 5 months of immunotherapy, marking the longest recorded occurrence of this phenomenon. This particular case of post-immunotherapy HPD achieved satisfactory results through targeted segment arterial chemoembolization, offering a potential approach for managing this side effect.

This case challenges the initial definition of HPD and has implications for the treatment of HPD, which had cautionary implications for the treatment of advanced cancers, particularly immunotherapy for cancer.

Molecular targeted therapy, antiangiogenic therapy, precision chemotherapy, radiation therapy, biotherapy, particle implantation therapy, and ICI have significantly advanced cancer therapy by showing different responses in different patients with different advanced tumor types (8–11). ICI have revolutionized systematic treatment for advanced solid tumors, with unprecedented survival benefits and tolerable adverse effects (2). The outcomes of other treatment options are generally only effective or ineffective. Only ICI can result in different response patterns because of the unique mechanisms of pharmacological action that have been reported in the literature (12–14). Some patients receiving immunotherapy present with typical responses, including a complete response (CR), a partial response (PR), stable disease (SD), or progressive disease (PD), but atypical patterns of response may occur in a subgroup of patients, including a delayed response (DeR), pseudoprogressive disease (PsPD), HPD, and a dissociated response (DR).

Currently, HPD is provisionally defined as occurring within two months of receiving immunotherapy with as an increase in tumor burden (7). HPD’s definitional criteria and biological basis are not agreed upon, which necessitates multicenter studies and collaborations (5). The enlargement of the lesion in this patient occurred approximately five months after immunotherapy and two weeks after the start of radiation therapy. The most likely complication to be considered is radiation pneumonitis. The main clinical symptoms of acute radiation pneumonitis are dry cough, dyspnea and low fever. What’s more, it usually occurs four to twelve weeks after radiotherapy and rarely occurs with low-dose radiation therapy (15). In addition, the lung puncture biopsy of the enlarged part of the lesion was confirmed squamous cell carcinoma. So, the diagnosis of radiation pneumonitis was ruled out.

The phenomenon of lesion growth becomes paradoxically accelerated after only two weeks of radiation therapy, and the increased part of the lesion was indicated squamous cell carcinoma without misdiagnosed by CT (16). From a logical point of view, this HPD is not the dominant role of radiation therapy. Firstly, the amount of radiation therapy was ten fractions only, which is not enough to cause HPD. Secondly, the dose of radiotherapy is only 2 Gy per fraction, also insufficient to cause HPD. Additionally, the duration of radiation therapy is two weeks only, making it unlikely for the tumor to double in size within that time frame. Combined with the specific details of the case and previous literatures on HPD, MDT believes that immunotherapy is the main factor causing HPD. And only HPD has this biological feature, and the tumor growth becomes paradoxically accelerated in a short time after immunotherapy (6).

Once HPD happens, it rarely has a good prognosis in previous literature (13). However, this case was again significantly alleviated by target segment arterial chemoembolization in this case. Arterial chemoembolization therapy has emerged as a pivotal treatment modality for some types of cancers in previous literature (17, 18). Target segment arterial chemoembolization involves the selective delivery of chemotherapeutic agents directly into the lung artery, followed by embolization to restrict the blood supply to the tumor, thereby enhancing the local concentration of the drug and minimizing systemic exposure. This method has received successful due to its ability to target tumors effectively while preserving surrounding healthy lung tissue.

### Limitations

As a case report, this study does have notable limitations. The single-case nature of the report means that the findings cannot be generalized to a larger patient population. A larger sample size or comparative analysis with other cases of HPD would be necessary to establish whether this patient’s response is typical or if chemoembolization should become a standard treatment approach for HPD. This study also fails to provide a clear explanation of the underlying biological mechanisms of HPD or how immunotherapy and radiation therapy might interact to trigger this phenomenon. Understanding these mechanisms would be critical for clinicians to better predict and manage HPD in patients. Furthermore, while the case shows significant tumor reduction following chemoembolization, the study lacks long-term follow-up data to assess whether the tumor remained stable or if further treatment was needed, which would have been valuable for assessing the durability of the response.

## Conclusion

In conclusion, this study is the first to present HPD after 5 months of immunotherapy, marking the longest recorded occurrence of this phenomenon. This particular case of post-immunotherapy HPD achieved satisfactory results through targeted segment arterial chemoembolization, offering a potential approach for managing this side effect.

## Data Availability

The original contributions presented in the study are included in the article/supplementary material. Further inquiries can be directed to the corresponding authors.
